# Study of Tunable Dielectric Permittivity of PBDB-T-2CL Polymer in Ternary Organic Blend Thin Films Using Spectroscopic Ellipsometry

**DOI:** 10.3390/polym15183771

**Published:** 2023-09-14

**Authors:** Laura Hrostea, Georgiana-Andreea Bulai, Vasile Tiron, Liviu Leontie

**Affiliations:** 1 Research Center on Advanced Materials and Technologies (RAMTECH), Department of Exact and Natural Sciences, Institute of Interdisciplinary Research, Alexandru Ioan Cuza University of Iasi, Bulevardul Carol I, Nr. 11, 700506 Iasi, Romania; vasile.tiron@uaic.ro; 2 Integrated Center of Environmental Science Studies in the North Eastern Region—CERNESIM, Department of Exact and Natural Sciences, Institute of Interdisciplinary Research, Alexandru Ioan Cuza University of Iasi, Bulevardul Carol I, Nr. 11, 700506 Iasi, Romania; georgiana.bulai@uaic.ro; 3 Faculty of Physics, Alexandru Ioan Cuza University of Iasi, Bulevardul Carol I, Nr. 11, 700506 Iasi, Romania

**Keywords:** polymer, ellipsometry, dielectric permittivity, photoconduction

## Abstract

The ellipsometric analyses reported in this paper present a novelty by bringing an in-depth optical investigation of some ternary organic blends. This study focuses on the tunability and control of the relative permittivity of active layers by varying the weight ratio of blended materials spin-coated as thin films. To investigate this, an extensive approach based on spectroscopic ellipsometry was conducted on ternary blend (D:A_1_:A_2_) thin films, involving a donor [D = chlorinated conjugated polymer (PBDB-T-2Cl)] and two acceptor materials [A_1_ = a non-fullerene (ITIC-F) and A_2_ = a fullerene (PCBM)]. The refractive index constitutes a key parameter that exposes insights into the feasibility of photovoltaic cells by predicting the trajectory of light as it transits the device. In this term, higher obtained refractive indexes support higher absorption coefficients. Notably, the dielectric constant can be rigorously tuned and finely calibrated by modest variations in the quantity of the third element, resulting in considerable modifications. Moreover, the inclusion of fullerene in blends, as the third element, results in a smooth topographical profile, further refining the surface of the film. From an electrical point of view, the ternary blends outperform the polymer thin films. The synergistic interaction of constituents emphasizes their potential to enhance solar conversion devices.

## 1. Introduction

Organic solar cells (OSCs) [1,2,3] have undergone significant advancements and progress over time, reaching the highest efficiency of up to 20% in 2022, recorded by [4] for a tandem solar cell (polymer:non-fullerene and perovskite layers). Simultaneously, heterojunction solar cells based on polymer:non-fullerene have begun a new trend with highly promising photovoltaic potential, achieving the highest efficiency of 19.3% in 2022 [5]. Traditionally, a typical OSC is based on a bulk heterojunction structure, composed of an electron donor material (conjugated polymer) and an electron acceptor material (fullerene or non-fullerene—NFA). In line with the advancements made in heterojunction-based photovoltaics and the potential of tandem solar cells, the concept of ternary solar cells has emerged. Ternary solar cells have garnered scientific interest due to their ease of fabrication and favorable optical performances. The current highest recorded efficiency for a ternary solar cell stands at 18.74%, achieved in 2023 [6].

Ternary organic solar cells (TOSCs) consist of a single bulk heterojunction based on either two donor materials and one acceptor or on one donor and two acceptor materials [7,8]. The donors are conjugated polymer materials, while the acceptors might be fullerenes or non-fullerenes. The success of fullerene materials is governed by a series of advantageous properties, such as high electron withdrawing capacity; the 3D charge transport provided by spherical geometry; and good miscibility with the donor material (polymer). The latter ensures the formation of an appropriate morphology that supports efficient charge carrier transport [9]. Unfortunately, the fullerenes are also characterized by some drawbacks that can hinder organic solar cell performances. For instance, fullerenes do not exhibit strong absorption in the visible range. The issue of the impossibility of tuning mechanisms is determined by the rigid structure of backbones, limiting the variation of bandgap and encumbering the chemical synthetization processes. On the one hand, the 3D ball shape favors, in fact, crystallization, but, on the other hand, also favors aggregations, thus reducing lifetime and stability. 

Since they were developed in 2015 [10], NFAs have exhibited remarkable potential as acceptor materials in solar cell applications. Compared to fullerene materials, NFAs are predisposed to chemical tuning processes, and in addition, they exhibit a broad absorption range and are easily and cheaply synthesized [11]. Working on the NFA chemical structure, the flexibility of use in a solar cell, over time, favored an increase in efficiency—surpassing the record of 18% reported in 2020 by Liu et al. [12]. In addition, the high achievement of these emerging material-based configurations is supported by a good matching with donor materials in the heterojunction blend with respect to energy level alignment [13].

With the multiple-component matrix adapted to the heterojunction structure [14,15], ternary solar cells can provide superior performances due to the complementarity of the materials. The broad spectral absorption, the compatible and cascade-like energy level alignment, the transport mechanism, and the enhanced morphology are just a few tremendous advantages of a ternary active layer to boost solar cell efficiency [7]. This method allows for the flexibility of selecting the most promising materials with complementary properties [8,15], while the role of the third component is critical, as it allows for numerous enhancements such as broadening the optical absorption spectra and facilitating charge carrier transport.

One noticeable metric that gains relevance within the broad spectrum of parameters, extending from macroscopic to nanoscopic features, comprising both extrinsic and intrinsic elements, that collectively determine the solar cell performance and feasibility, is the dielectric permittivity. This complex parameter is directly related to the refractive index able to define the light trajectory: a major source of energy loss is photon reflection from the layer caused by a rapid change in the refractive index between all the device layers [16,17]. The dielectric constant of a material can be determined by using two well-known methods: reflection and transmission measurements and ellipsometry. In order to calculate the complex permittivity, based on the first method, both reflection and transmission are needed in the former, which may represent a huge drawback in the case of opaque surfaces, requiring supplementary calculation. Analyzing the change in the polarization state of light once reflected on a thin film, spectroscopic ellipsometry easily provides data to calculate the real and the imaginary part of the dielectric function, depending on the amplitude ratio and phase difference [18]. 

Spectroscopic ellipsometry (SE) can be used to analyze almost any transparent thin film and is able to measure layers as thin as the wavelength of the probing light itself down to less than a single atomic layer, beyond the thickness limits of equivalent ellipsometry- and reflectometry-based techniques, and is especially helpful for ultra-thin film applications (100 nm) [19]. Spectroscopic ellipsometry provides extremely accurate, reproducible measurements of the dielectric properties of a material, including complex refractive index and dielectric function tensor, and optical constants, in addition to film thickness [20]. Ellipsometry is based on the physical phenomenon in which the linearly polarized light is reflected off of a surface in a different manner depending on the surface electric field (parallel or perpendicular to the incidence plane); the output elliptical polarization state is then analyzed by measuring the ellipsometric quantities *Ψ* (amplitude ratio) and *Δ* (phase difference between reflected and incident light) [21]. The tunability and the control of the real part of the dielectric constant, which corresponds to the relative dielectric constant (*ε_r_*), are of huge importance in improving the photovoltaic performance of a device [22]. 

The goal of the current study is to highlight a significant development, specifically the possibility of controlling the dielectric permittivity in thin films used as active layers in solar cells. This study establishes new ground in the field by analyzing both binary and ternary blend films using a comparative methodology. The donor material in the binary-based films is PBDB-T-2Cl, and the acceptor materials can be ITIC-F or PCBM, while the investigated ternary blend films involve the same donor material along with both acceptor materials (D:A_1_:A_2_ = PBDB-T-2Cl:ITIC-F:PCBM). What sets this study apart is its extensive analysis of ellipsometric parameters, focusing on the synergistic effects of the polymer, non-fullerenes, and fullerenes from optical and morphological perspectives. Furthermore, the research explores the possibility of tuning the dielectric constant, offering a novel contribution to the field.

## 2. Materials and Methods

These materials were purchased from Sigma Aldrich, Steinheim, Germany: a conjugated polymer, Poly[(2,6-(4,8-bis(5-(2-ethylhexyl-3-chloro)thiophen-2-yl)-benzo [1,2-b:4,5-b′]dithiophene))-alt-(5,5-(1′,3′-di-2-thienyl-5′,7′-bis(2-ethylhexyl)benzo [1′,2′-c:4′,5′c′]dithiophene-4,8-dione)], in short PBDB-T-2Cl, having a molar weight of 50,000–100,000 g/mol; a non-fullerene,3,9-bis(2-methylene-((3-(1,1-dicyanomethylene)-6,7-difluoro)-indanone))5,5,11,11tetrakis(4-hexylphenyl)-dithieno [2,3-d:2′,3′-d′]-s-indaceno [1,2-b:5,6-b′]dithiophene, in short ITIC-F, with a purity of 99%; and a fullerene, [6,6]-Phenyl-C61-butyric acid methyl ester (>99.5% purity), in short PCBM.

Chlorobenzene was employed as the solvent, and separate solutions of each ingredient material were prepared at a concentration of 20 mg/mL by magnetic stirring for two hours at 60 °C. The resulting solutions were then combined in different weight ratios to create the desired blends: non-fullerene-based blend (polymer:non-fullerene = 1:1.4), fullerene-based blend (polymer:fullerene = 1:1.4), and ternary blends [polymer:non-fullerene:fullerene = D:A_1_:A_2_ = 1:1.2:0.2 and 1:1:0.4, respectively]. The blends were spun onto glass substrates for 40 s at 750 rotations per minute to deposit them as thin films, after cleaning the glass for 10 min in an ultrasonic bath with acetone, ethanol, and detergent. The samples were dried at 100 °C for 10 min after deposition. The entire preparation process was carried out in a clean room with a laboratory hood that provided a normal environment, and the samples were kept in the dark. According to the decrease in the non-fullerene acceptors (NFAs) ratio, the samples in this study are labeled as follows: ITIC-F-based thin film (1:1.4:0), “A” Ternary thin film (1:1.2:0.2), “B” Ternary thin film (1:1:0.4), and PCBM-based thin film (1:0:1.4).

The thickness of the samples was determined by profilometry using a DektakXT Stylus profilometer (Bruker France S.A.S., Wissembourg, France), as well as by spectroscopic ellipsometry. The optical absorption was analyzed using a TEC5 spectrophotometer, while the refractive index and extinction coefficient were determined by spectroscopic ellipsometry in a spectral range from 250 to 1100 nm using a Horiba Jobin Yvon UVVISELTM ellipsometer (Horiba Jobin Yvon, Longjumeau, France), equipped with a 75 W high luminance discharge Xe lamp. All the ellipsometric spectra were recorded at room temperature, at an incident angle of 70°. The ellipsometric models, developed for the sample simulation, were carried out using DeltaPsi 2.7 software of Horiba Jobin Yvon, which allowed the fit of the experimental amplitude ratios, *Ψ*, and the phase differences, Δ. Atomic Force Microscopy (AFM) images were recorded using a NT-MDT Solver Pro-M system (from NT-MDT, Moscow, Russia). The measurements were performed in non-contact mode, at ambient temperature, using a SiN cantilever (NSC21 from Mikromasch, Tallinn, Estonia). The root mean square roughness (*R_RMS_*) of the samples was estimated for a 5 × 5 µm^2^ scanned area using Nova software (version 1.0.26.1443) from NT-MDT. The photoconduction processes were emphasized by measuring the resistance under light and dark conditions using a white light source at room temperature.

## 3. Results

The study is focused on the identification of ellipsometric parameters of individual constituent materials (PBDB-T-2Cl, ITIC-F, and PCBM) and of the organic films (binary and ternary, as previously mentioned), using spectroscopic ellipsometry [23].

**Figure 1:**
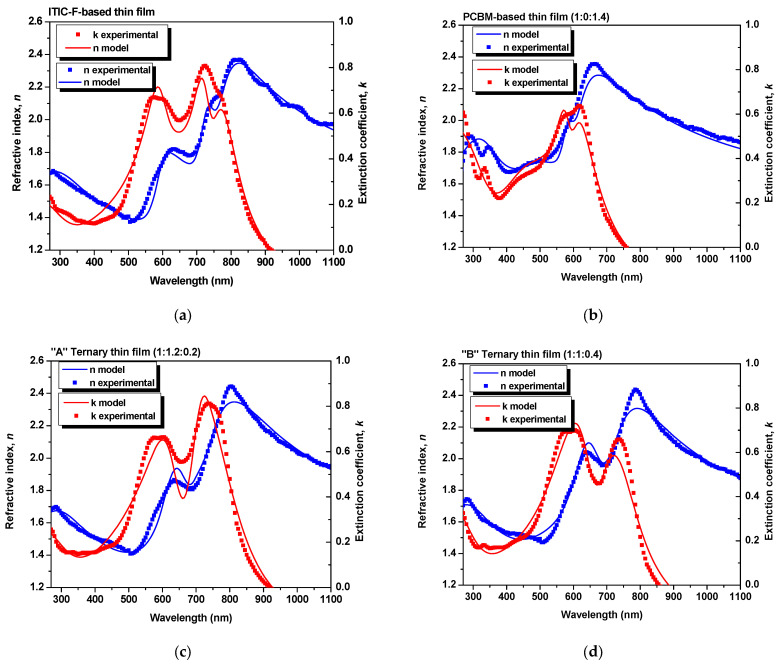
Refractive index and extinction coefficient—a good match between the experimental data and simulated data using spectroscopic ellipsometry of the binary and ternary organic blend thin films, for (**a**) ITIC-F-based thin film, (**b**) PCBM-based thin film, (**c**) “A” Ternary thin film and (**d**) “B” Ternary thin film.

Figure 1 shows the experimental data and the simulated values for the refractive index and extinction coefficient, as “global” parameters. The experimental data were fitted using a model based on a bi-layered structure [23], involving glass as a substrate and an organic film. Therefore, the refractive index of the layers quantifies the modification in terms of the speed of light inside the layer with respect to the speed of light in a vacuum. At the same time, the extinction coefficient characterizes the absorption losses at the layer level.

The layers were simulated by the New Amorphous Dispersion formula of three oscillators [21,24,25], determining the refractive index [Formulas (7) and (8)] and the extinction coefficient [Formula (9)]:

(1) $$n=n_{\infty }+\frac{B_{j}(\omega -\omega_{j})+C_{j}}{(\omega -\omega_{j}{)}^{2}+\Gamma_{j}^{2}}$$

(2) $$\left\{\begin{array}{c} B_{j}=\frac{f_{j}}{\Gamma_{j}}\cdot [\Gamma_{j}^{2}-(\omega_{j}-\omega_{g}{)}^{2}]\\ C_{j}=2f_{j}\cdot \Gamma_{j}\cdot (\omega_{j}-\omega_{g}) \end{array}\right.$$

(3) $$k=\left\{\begin{array}{l} \frac{f_{j}\cdot (\omega -\omega_{g}{)}^{2}}{(\omega -\omega_{j}{)}^{2}+\Gamma_{j}^{2}},\;\omega >\omega_{g}\\ 0,for\;\omega <\omega_{g} \end{array}\right.$$

in the expressions of which the parameters presented in Table 1 are involved. In the above equations, *n*(*ω*) and *k*(*ω*) denote the refractive index and the extinction coefficient, respectively, *Γ_j_* is the widening parameter of the absorption peak, *f_j_* represents the amplitude of the extinction coefficient, *ω_j_* defines the energy for the maximum extinction coefficient, while *ω_g_* denotes the energy for the minimum extinction coefficient. The accuracy of the model is given by *χ*^2^, which quantifies the goodness of the fit (*χ*^2^ < 10) [20,26].

**Figure 2:**
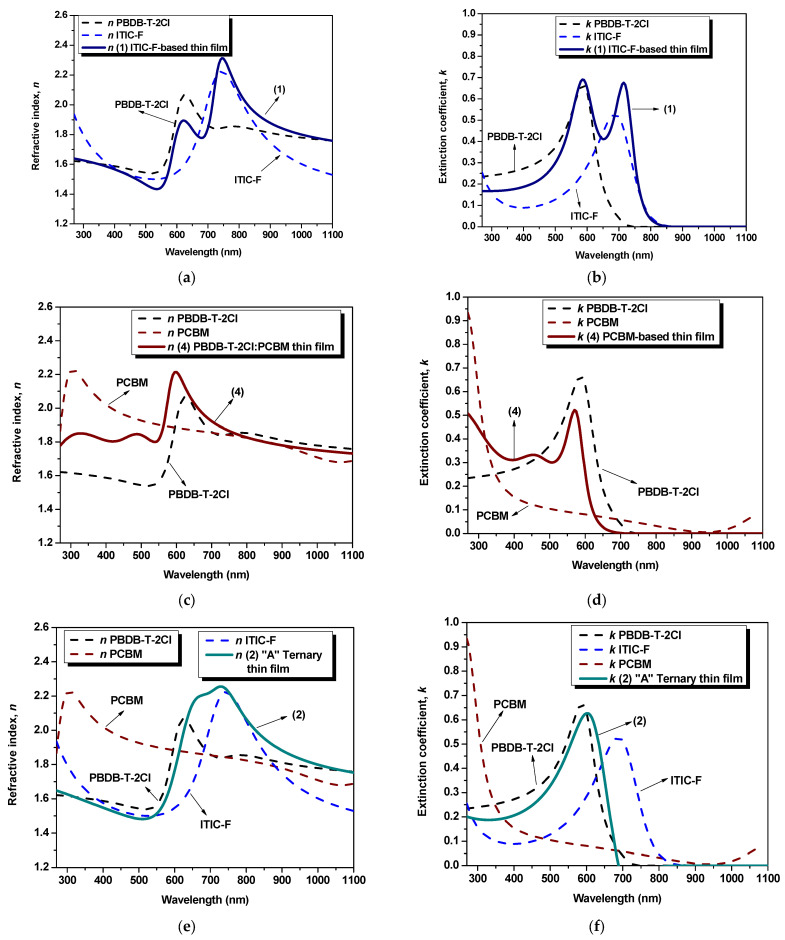

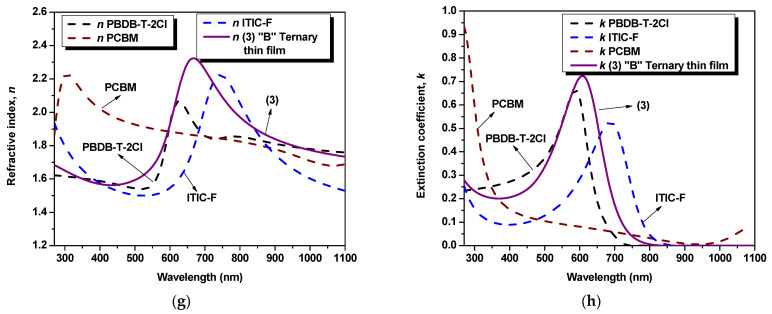
Spectral dependencies of refractive index [(**a**,**c**,**e**,**g**)] and extinction coefficient [(**b**,**d**,**f**,**h**)] of individual layers.

To validate the results obtained from the simulations, as one can see in Table 2, the values of the layers’ thickness, obtained by profilometry (as direct measurements) and by ellipsometry, were compared, and the parameter *χ*^2^ (where *χ*^2^ < 10) was also taken into account, which quantifies the matching of the ellipsometric experimental data with those generated by simulation [27]. As obtained by extracting from the models, the ellipsometric parameters for each blend thin film and for the constituent materials thin films, the dispersion curves of refractive index and extinction coefficient are described in Figure 2.

**Figure 3:**
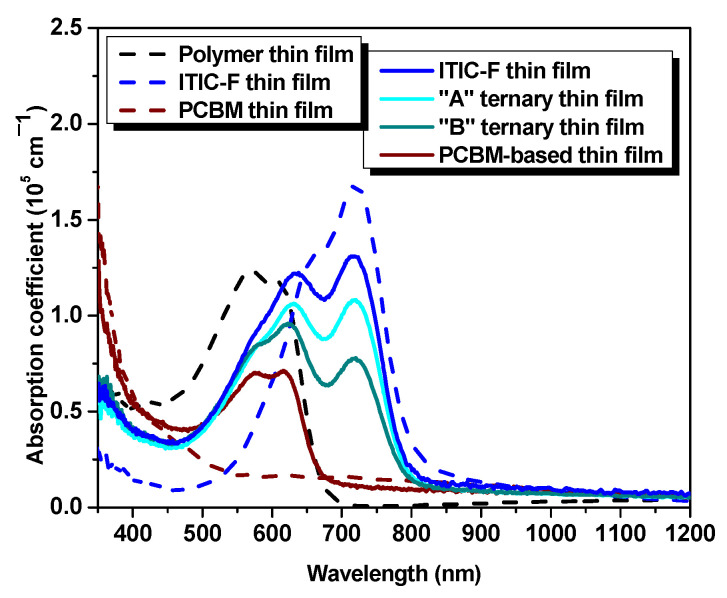
Absorption coefficients of single-material-solution- and blended-material-solution-coated thin films.

The refractive index denotes a quantifiable measure of the variation in the speed of light as it traverses diverse media. Subsequently, the refractive index of a layer, constituted by multiple materials, undergoes changes due to several factors. These encompass inter-particle interactions, the refractive indices of the composite constituents, the concentration and dispersion phenomena, and the topographical assets and microstructural properties inherent to the layer. Thirdly, the refractive index constitutes a key parameter that delivers insights into the feasibility of photovoltaic cells by predicting the trajectory of light as it transits the device, favoring the photon absorption or not, encompassing transitions between varying refractive indices across individual layers. Concerning the influence of the refractive index on optical properties (absorption/transmission), a higher value signifies higher absorption coefficients. The maximum refractive index values, n_max_, of the materials are n_max_^polymer^ = 2.06 at 625 nm wavelength, lower than its precursor PBDB-T, identified by Kerremans (2020) and Harillo Banos (2020) as being equal to 2.4 [28] and 2.25 [29], respectively; n_max_^PCBM^ = 2.22 at 305 nm wavelength; and n_max_^ITIC-F^ = 2.00 at 745 nm wavelength, lower than 2.4 according to [28] at the same wavelength and even lower than its precursor ITIC, n^ITIC^ = 3.35 [29]. Thus, the refractive index of the blend thin films shows an increase with respect to the value of each individual material thin film and a continuous redshift once the non-fullerene amount is increased for both binary and ternary blend thin films. For the non-fullerene-based film (Figure 2a), n_max_ = 2.31 at 745 nm wavelength, notably lower than the binary blend based on PBDBT:ITIC (their precursors), where the refractive index is 2.6 at 745 nm wavelength [28]. At the same time, the fullerene-based blend thin film (Figure 2c) exhibits a maximum refractive index of 2.21 at 595 nm wavelength. Moreover, in Figure 2e, in the case of the “A” ternary blend thin film (1:1.2:0.2), the highest refractive index [n_max_^“A”^ = 2.25] is identified at 725 nm wavelength, while the highest n, for the “B” ternary blend thin film (1:1:0.4) is slightly higher than the previous one [n_max_^“B”^ = 2.32] and shifted to 665 nm wavelength, as can be seen in Figure 2g. Thus, the highest peak, for both binary and ternary blends, is a result of the presence of both the non-fullerene and the polymer, as a linear combination of their respective contribution. In this case, varying the amount of fullerene (e.g., by increasing it), the dispersion spectra of n for the ternary blends show the fingerprints of the fullerene: modifying n and blue-shifting the peak wavelength position. Regarding the extinction coefficient, k, the samples exhibit a similar behavior as shown in the absorption spectra [Figure 3]. The non-fullerene-based film (Figure 2b) describes a shape that combines the behavior of constituent materials, displaying two peaks: at 605 nm wavelength (k_1_ = 0.62), specific to the polymer, and at 715 nm wavelength (k_2_ = 0.67), specific to the non-fullerene. These values are lower compared to their precursor blends [28,30]. Meanwhile, the extinction coefficient, k, in the case of the fullerene-based blend (Figure 2d), is lower than that of the polymer (k^polymer^ = 0.66 at 590 nm wavelength), exhibiting a value of 0.52 at 570 nm wavelength.

The influence of the non-fullerene and fullerene components can be observed in the behavior of the ternary blends in Figure 2f,h. It is evident that the blends exhibit similar trends, with a higher peak when the fullerene content in the ternary blend is increased. This observation suggests that the fullerene component has a significant impact on the dielectric properties of the blends. By comparing the parameters of the blends with those of the individual materials, a distinct fingerprint can be observed, indicating the contribution of each constituent in the mixtures. This suggests that the blending process has a noticeable effect on the dielectric properties of the samples.

The experimental results analyzed by ellipsometric technique and the calculated ellipsometric parameters are in agreement with the direct measurements of optical properties (such as absorption coefficient—Figure 2) and with the thickness obtained by profilometry (Table 2).

The optical absorption characteristics of the samples are analyzed in Figure 3, via the absorption coefficient. The polymer is harvesting the visible range photons in the spectral range of approx. 450–690 nm. In contrast, the acceptor materials spectra are redshifted relative to the polymer absorption spectrum, in the 500–850 nm range for non-fullerene, respectively blueshifted in the 350–500 nm range in the case of fullerene. As can be inferred from Figure 3, the optical absorption spectrum of the non-fullerene-based film is widened, lying between 460 and 820 nm, being shifted (≈80 nm) to the near-infrared range with respect to the polymer absorption spectrum. The absorption spectrum of the fullerene-based thin film exhibits a 60 nm spectral range narrowing of the polymer optical potential and a slight increase in the absorption coefficient amplitude in the near-infrared wavelength range. It is obvious the spectral difference of the studied acceptor materials and, at the same time, the presence of the fullerene, as a third component, in the polymer:non-fullerene host matrix slightly improve the optical absorption. These facts highlight the enhancement of the optical properties in terms of absorption of the ternary blends and represent a step forward to an efficiency boost for further applications, due to the complementarity in optical absorption of the three workhorses involved in the mixtures.

**Figure 4:**
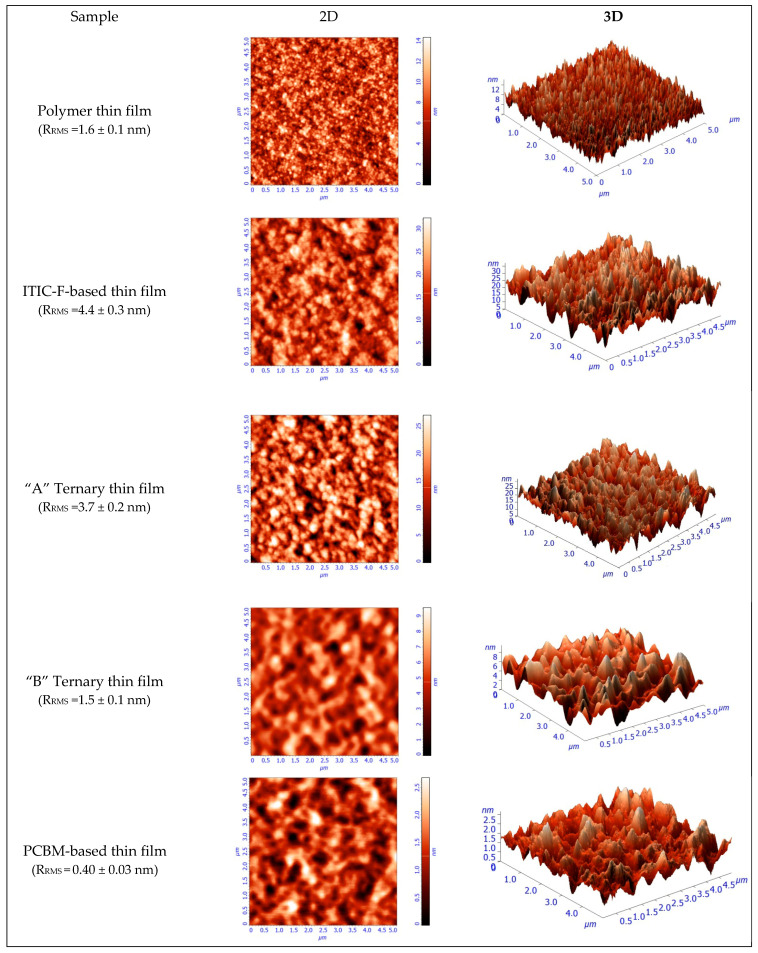
AFM micrographs illustrating morphological changes of the ternary organic blend thin films.

Moreover, in terms of topography, as investigated by atomic force microscopy, the samples display well-mixed components and a very smooth surface. Figure 4 presents 5 × 5 µm^2^ images in 2D and 3D representations, obtained by AFM for the polymer thin film and for all the four blend layers. In addition, the polymer layer exhibits a 1.6 nm roughness, as shown in Figure 4. From this value, the *R_RMS_* is increased by adding the non-fullerene to 4.4 nm and slowly decreased while increasing the amount of fullerene, as a third component, to 3.7 and 1.5 nm, respectively. This is due to a higher miscibility of the fullerenes and non-fullerenes in the polymer matrix, and the variation of the roughness leads to the variation in the layer thickness, as can be seen in Table 2. The use of NFAs generally leads to higher roughness compared to fullerene-based blend films. The increased roughness results in a larger active surface area, which enhances absorption, as evident in the absorption coefficient shown in Figure 3. Additionally, good miscibility between the materials reduces the driving force for phase separation, resulting in smaller impurity domains that support efficient electron-hole dissociation.

**Figure 5:**
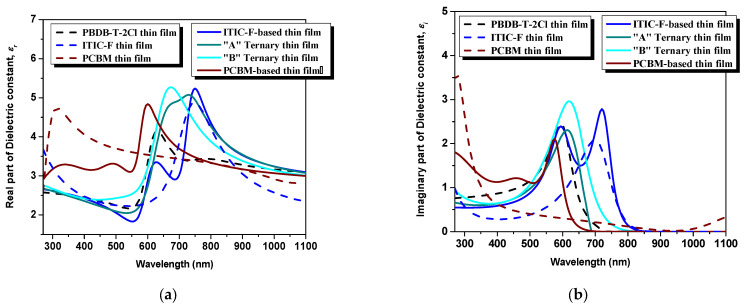
Dielectric constant from ellipsometry analysis: real part (**a**) and imaginary part (**b**).

The tunability and the possibility of control of the real part of the dielectric constant (relative dielectric constant) are known as key factors for superior photovoltaic performances of a device [22]. A low ε_r_ in organic semiconductors can result in increased Coulomb interactions between charge carriers, which can have a negative impact on the separation of the photogenerated charge carriers and can promote the recombination processes. This leads to the necessity of increasing the ε_r_ value. A higher ε_r_ facilitates faster charge transfer at the donor–acceptor interface, leading to a low offset of the potential photovoltaic device and a reduction in recombination rate, which will further reduce the bias dependence of the photocurrent and will increase the fill factor. In summary, the exciton separation rate can be increased while lowering the electron-hole binding energy, and the geminate recombination rate can be reduced if the ε_r_ value is increased. As can be seen in Figure 5a (dotted lines), the pristine polymer thin film presents the lowest maximum value of ε_r_ with respect to the non-fullerene and the fullerene: ε_r_^PBDB-T−2C l^= 4.1 at 635 nm wavelength versus ε_r_^ITIC-F^ = 4.8 at 745 nm, respectively ε_r_^PCBM^ = 4.72 at 310 nm. The dielectric environment changes once the acceptor materials are mixed in the polymeric matrix by empowering its character: ε_r_^PBDB-T−2Cl:ITIC-F^ = 5.2 at 750 nm versus ε_r_^PBDB-T−2Cl:PCBM^ = 4.83 at 600 nm. Regarding the ternary blends, for the “A” blend, where the PCBM amount is lower, the maximum peak, ε_r_*^“^*^A*”*^ = 5.07 at 730 nm, is higher than each pristine material presenting a shoulder from 660 to 730 nm, practically in-between the spectral ranges of the constituents. The highest ε_r_ is ascribed to the “B” blend thin film, ε_r_*^“^*^B*”*^ = 5.2 at 670 nm wavelength, providing the most dielectric medium of all the blends. These changes in dielectric constant values are attributed to the presence of local dipoles at the donor-acceptor interfaces. The fullerene, acting as the third component, serves as a binder in the polymer:non-fullerene host matrix, promoting intimate interactions between the donor and acceptor materials. This intimate interaction facilitates the formation of charge transfer states, which are crucial for efficient charge separation and transport in organic solar cells. The presence of fullerene in the ternary blend enhances the charge transfer process and contributes to the overall improvement of the photovoltaic performance [31,32].

**Figure 6:**
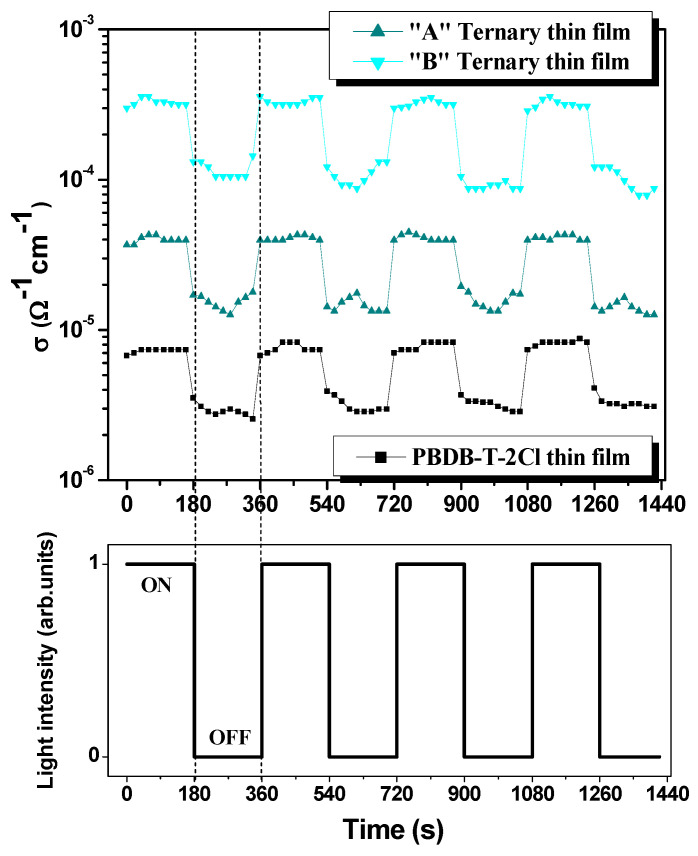
Light effect on ternary blend thin films.

Concerning the imaginary part of the dielectric constant, which emphasizes the loss mechanisms (e.g., conductance), as one can see in Figure 5b, the highest peak of ε_i_ = 2.96 at 620 nm can be attributed to the “B” ternary blend thin films. In this case, in order to highlight the electrical conductance of the ternary blends and, additionally, the film reaction to light, regarding the photoconductivity performances, several dark-light steps were applied, as visible in Figure 6.

Figure 6 describes the electrical conductivity behavior of pristine polymer and ternary blend thin films in a light–dark step-like cycle. Firstly, the polymer exhibits the lowest conductivity ($\sigma_{dark}^{polymer}\approx 2.7\times 10^{-6}\;\Omega^{-1}{cm}^{-1}$ and $\sigma_{light}^{polymer}\approx 7.3\times 10^{-6}\;\Omega^{-1}{cm}^{-1}$), followed by the “A” ternary thin film (where the weight ratio D:A_1_:A_2_: PBDB-T-2Cl:ITIC-F:PCBM = 1:1.2:0.2) whose conductivity is $\sigma_{dark}^{”A”}\approx 1.4\times 10^{-5}\;\Omega^{-1}{cm}^{-1}$ and $\sigma_{light}^{”A”}\approx 3.9\times 10^{-5}\;\Omega^{-1}{cm}^{-1}$. The highest conductivity value is reached by the “B” ternary blend thin film (D:A_1_:A_2_ = 1:1:0.4), as follows: $\sigma_{dark}^{”B”}\approx 1.04\times 10^{-6}\;\Omega^{-1}{cm}^{-1}$ and $\sigma_{light}^{”B”}\approx 3.2\times 10^{-6}\;\Omega^{-1}{cm}^{-1}$, which is correlated with the highest value of the imaginary part of the dielectric constant. To sum up this point of observation, the electrical conductivity is enhanced with roughly one order of magnitude for each 0.2 increase in the weight ratio of the fullerene. Secondly, the response to light of the polymer and the blends is highly pronounced. Compared to dark conditions, the effect of light can be quantified by an almost three times increase in conductivity for each film, even slightly higher in the case of the “B” ternary film. Moreover, under illumination, the electrical behavior is more stable, while under dark conditions, some inertial processes take place, determining a fluctuating behavior.

## 4. Conclusions

This study emphasizes the optical performances of the individual materials and the synergistic effects observed in the ternary. The in-depth ellipsometric analysis allows the simulation of experimental structures and the identification of optical parameters (such as refractive index, extinction coefficient, and dielectric constant). The dispersion curves of the refractive index and extinction coefficient for single-material layers become visible fingerprints in the binary and ternary dispersion curves, as a result of the optical complementarity of the precursor materials. The possibility of tuning the dielectric constant of the thin films by varying the weight ratio was revealed, while, in terms of conductivity, a visible increase of one order of magnitude is obtained for each 0.2 step of increasing the fullerene weight ratio. The photoconduction processes are more pronounced in the case of D:A_1_:A_2_ = 1:1:0.4 weight ratio.

The compatibility and the performances of the organic materials in the blends were also investigated from the optical and morphological point of view, and the influence of the weight ratio of constituents in organic ternary blends was discussed. The layers present a very smooth surface at nanoscale, and the weight ratio of the third component (the fullerene) has an important role in the enhancement of the roughness. From the optical point of view, the ternary blend thin films exhibit an excellent widening of the solar absorption spectrum, from 460 nm to 820 nm, due to the complementarity of the constituent materials from a light-harvesting perspective. Furthermore, regarding their potential as active layers in organic solar cells, the presence of the non-fullerene had a significant effect of broadening the absorption spectrum, while the third component (fullerene) contributed to improvements in optical, morphological, and electrical properties. These findings highlight the promising characteristics of ternary blend systems for applications in organic solar cells.

## Figures and Tables

**Table 1:** Ellipsometric parameters for binary and ternary blend thin films.

Sample	Dispersion Formula	Specific Parameters					Thickness (nm)	$\chi^{2}$
		$n_{\infty }$	$\omega_{g}$ (eV)	$f_{j}$	$\omega_{j}$ (eV)	$\Gamma_{j}\;$(eV)		
ITIC-F-based thin film (1:1.4:0)	3 × NA	1.68	1.41	0.05	1.70	0.09	142 ± 1	5.69
				0.03	2.08	0.16		
				0.13	0.05	2.15		
“A” Ternary thin film (1:1.2:0.2)	3 × NA	1.47	1.40	−0.03	1.70	−0.12	145 ± 1	5.02
				0.11	1.94	0.22		
				0.28	2.77	5.33		
“B” Ternary thin film (1:1:0.4)	3 × NA	1.38	1.46	−0.03	2.27	−0.54	138 ± 1	3.09
				0.15	1.93	0.23		
				0.07	5.67	2.10		
PCBM-based thin film (1:0:1.4)	3 × NA	1.46	1.69	0.03	2.13	0.11	119 ± 1	3.75
				0.03	2.55	0.40		
				0.18	3.84	1.84		
PBDB-T-2CL thin film	3 × NA	1.65	1.66	0.12	1.67	−0.18	202 ± 1	8.16
				0.08	2.04	0.15		
				0.01	0.59	0.0.3		
ITIC-F thin film	3 × NA	0.97	1.38	−0.15	1.61	−0.37	127 ± 5	2.69
				0.17	1.71	0.18		
				0.10	6.38	1.28		
PCBM thin film	3 × NA	1.66	0.96	−0.49	1.06	0.18	91 ± 1	2.07
				0.62	1.00	0.14		
				0.02	4.50	0.66		

**Table 2:** Thickness of the sample by profilometry vs. ellipsometry as individual material films and as blend films.

Sample	Profilometry	Ellipsometry
	Thickness (nm)	Thickness (nm)
PBDB-T-2Cl thin film	203 ± 5	188 ± 1
ITIC-F thin film	115 ± 2	112 ± 3
PCBM thin film	90 ± 2	87 ± 1
ITIC-F-based thin film (1:1.4:0)	128 ± 3	142 ± 1
“A” Ternary thin film (1:1.2:0.2)	140 ± 4	140 ± 1
“B” Ternary thin film (1:1:0.4)	127 ± 2	135 ± 1
PCBM-based thin film (1:0:1.4)	108 ± 3	119 ± 1

## Data Availability

Data will be made available on request.
